# Analysis of the timing of endoscopic treatment for esophagogastric variceal bleeding in cirrhosis

**DOI:** 10.3389/fmed.2022.1036491

**Published:** 2022-12-01

**Authors:** Kaini Wu, Yunfeng Fu, Zixiang Guo, Xiaodong Zhou

**Affiliations:** Department of Gastroenterology, Digestive Disease Hospital, The First Affiliated Hospital of Nanchang University, Nanchang, China

**Keywords:** cirrhosis, esophagogastric variceal bleeding, endoscopy, timing, risk factor

## Abstract

**Background:**

Existing guidelines recommend endoscopic treatment within 12 h or 12–24 h for patients with esophagogastric variceal bleeding (EGVB) in cirrhosis. In addition, research findings on the optimal time for endoscopy are inconsistent.

**Aim:**

The aim of this study was to investigate the relationship between the timing of endoscopy and clinical outcomes in cirrhotic patients with EGVB and to analyze the risk factors for the composite outcomes after endoscopic treatment.

**Methods:**

From January 2019 to June 2020, 456 patients with cirrhotic EGVB who underwent endoscopy were matched by a 1:1 propensity score. Finally, 266 patients were divided into two groups, including 133 patients within 12 h (urgent endoscopy group) of admission and after 12 h (non-urgent endoscopy group). Baseline data and clinical outcomes were compared. Logistic regression model analysis was used to determine risk factors for 30 days rebleeding and mortality.

**Results:**

In 266 patients, the overall 30 days rebleeding rate and mortality were 10.9% (*n* = 29) and 3.4% (*n* = 9), respectively. Patients who underwent endoscopic treatment within 12 h had significantly higher 30 days rebleeding outcomes than those who underwent treatment beyond 12 h (15 vs. 6.8%, *p* = 0.003). However, 30 days mortality did not differ significantly between the two groups (3 vs. 3.8%, *p* = 0.736). Logistic regression analysis showed that age and shock on admission were independent risk factors for the composite outcome of 30 days rebleeding and mortality in patients with EGVB.

**Conclusion:**

The 30 days rebleeding rate in patients with cirrhotic EGVB treated with urgent endoscopy was significantly higher than that in patients treated with non-urgent endoscopy, but there was no significant difference in 30 days mortality.

## Introduction

Esophagogastric variceal bleeding (EGVB) is one of the most common medical emergencies of liver cirrhosis, with an annual incidence of about 12–15% (1) and a mortality rate of 20–25% (2, 3). Several therapies, including antibiotics, vasoactive medications, endoscopic methods, and interventional radiology treatments, are currently used to treat EGVB (4–6). In particular, endoscopic therapy can not only identify the site and source of bleeding and stop the bleeding urgently but also prevent rebleeding. Although some guidelines, such as the American Association for the Study of Liver Diseases (AASLD) and the European Association for the Study of the Liver (EASL), suggest that endoscopic therapy of EGVB should be completed within 12 h, some Chinese guidelines suggest 12–24 h (7–9). Furthermore, these controversial recommendations on the optimal timing of endoscopy are based on expert opinions, rather than clinical studies.

Many previous observational studies have shown that endoscopy timing was not related to rebleeding and mortality in patients with EGVB (10, 11). However, a retrospective study of patients with hematemesis symptoms revealed that urgent endoscopy in cirrhotic patients with acute EGVB did decrease rebleeding rates and mortality (12). A recent study suggested that urgent endoscopy is detrimental to the prognosis of bleeding patients (13). Data from these studies are few and inconsistent, and the optimal timing of endoscopy remains uncertain.

The purpose of this study was to determine the relationship between the timing of endoscopic treatment and clinical outcomes in cirrhotic patients with EGVB. In addition, we aimed to examine risk factors for the composite outcome of rebleeding and mortality after endoscopic treatment.

## Materials and methods

### Patients

From January 2019 to June 2020, patients with cirrhosis suspected of EGVB were admitted to our hospital. The inclusion criteria were (1) age of ≥18 years and (2) confirmed cirrhosis with esophageal or gastric variceal bleeding. The exclusion criteria were (1) serious organ dysfunction diseases: heart failure, kidney failure, etc.; (2) malignancy other than liver malignancy; and (3) upper gastrointestinal bleeding other than varicose bleeding in cirrhosis. Diagnostic criteria for EGVB in cirrhosis include active variceal bleeding or no obvious bleeding foci based on cirrhosis but variceal thrombosis head confirmed by endoscopy (7).

### Data collection

Two investigators (W.K.N. and F.Y.F.) independently reviewed the medical records of the included patients to collect demographic, laboratory, clinical, endoscopic, and therapeutic data. We calculated the Child-Pugh score, MELD score, and Glasgow-Blatchford score from medical data.

### Endoscopic procedure

When a patient with cirrhosis suspected of EGVB arrives at the hospital, the receiving physician immediately administers adequate fluid resuscitation, prophylactic antibiotics, proton-pump inhibitors, and vasoactive drugs (terlipressin, somatostatin, and octreotide) (8). The timing of endoscopy is determined by the gastrointestinal endoscopist, taking into account the patient’s age, the presence of comorbidities such as renal failure or cardiopulmonary disease, the presence of hepatic encephalopathy, hemodynamic status, and laboratory abnormalities including severe anemia and coagulopathy. On weekday night shifts or weekends, the hospital still has a gastrointestinal endoscopist with expertise in endoscopic hemostasis and a support staff skilled in the use of endoscopic equipment on call. Standard video endoscopes (GIF-Q260; Mount Olympus, Tokyo, Japan) are used to perform the treatment. We performed endoscopic variceal ligation (EVL) with standard ligating devices (Sumitomo Bakelite, Tokyo, Japan), endoscopic variceal obturation by injecting a tissue adhesive (Fu Aile, Beijing, China) with N-butyl cyanoacrylate mixed with N-octyl cyanoacrylate, and endoscopic injection sclerotherapy with lauromacrogol (Tianyu Pharmaceutical, Shanxi, China).

### Definitions

The endoscopic time was defined as the interval between admission and the start of the endoscopy. An endoscopy performed within 12 h was considered urgent endoscopy, and an endoscopy performed over 12 h was considered non-urgent endoscopy. Successful hemostasis was defined as the absence of active bleeding within 72 h after bleeding control. Rebleeding was defined as hematemesis, hematochezia, or melena with changes in laboratory tests (hemoglobin decreased to >2 g/dl within 24 h) or vital signs (systolic blood pressure decreased to <90 mmHg or heart rate increased to > 100 beats/min) and had to be confirmed by endoscopy. The 30 days composite outcome was defined as the composite of 30 days rebleeding and mortality.

### Outcome assessment and follow-up

The primary outcome was 30 days rebleeding. The secondary outcomes were 30 days mortality, the incidence of rescue therapy, the total number of transfused red blood cell units, the number of transfusion products, and the length of hospital stay.

The efficacy of the patients after endoscopic treatment (rebleeding, postoperative survival time, and cause of death if possible) was followed up. The investigators (W.K.N. and G.Z.X.) had access to outpatient or inpatient information and followed up by telephone. If telephone contact was unavailable or refused by patients, the time of the last visit was considered the time of loss to follow-up.

### Statistical analysis

Data were analyzed using SPSS version 26 (IBM, Armonk, NY, USA). R version 4.2.1 (The R Foundation for Statistical Computing, Vienna, Austria) was used to draw graphs. Propensity score matching (PSM) analysis was based on the following variables: age, sex, hematemesis, etiology of cirrhosis, liver cancer, infection, portal vein emboli, ascites, hepatic encephalopathy, systolic blood pressure on admission, heart rate, laboratory tests, treatment methods, antibiotic use, Child-Pugh score, MELD score, and Glasgow-Blatchford score. The 1:1 nearest-neighbor matching method was used for the endoscopic treatment of EGVB patients with cirrhosis, and 133 matched pairs were obtained in both groups.

Categorical variables were shown as quantities and percentages (%). Pearson’s chi-square test or Fisher’s exact test was used for the comparison between the groups. Continuous variables were expressed as mean ± standard deviation (X̄ ± s). Statistical differences between the groups were compared using Student’s *t*-test or Mann–Whitney U test. The value of *p* < 0.05 was considered statistically significant. The cumulative 30 days survival rates after acute variceal bleeding were estimated by the Kaplan–Meier method, and differences between the curves were compared by the log-rank test. The univariate logistic regression model was used to screen variables with *p* < 0.05. Multivariate analysis was performed using a binary logistic regression model. The odds ratio (OR) and 95% confidence interval (CI) were calculated.

## Results

### Patient characteristics

The basic characteristics of 456 patients included in the study are shown in Table 1. There were 191 patients (male/female = 143/48, mean age 53.0 ± 12.8 years) in the urgent endoscopy group and 265 patients (male/female = 190/75, mean age 53.1 ± 11.4 years) in the non-urgent endoscopy group. However, the baseline characteristics such as hematemesis, laboratory indicators, scores, and treatments differed between the two groups. The 1:1 nearest-neighbor matching method was performed in 456 patients with EGVB. In the end, the baseline data of 266 patients were not statistically different (*p* > 0.05), as shown in Table 2.

**TABLE 1:** Baseline characteristics of patients in urgent (≤12 h) and non-urgent (>12 h) endoscopy before propensity score matching (PSM).

Characteristics	Urgent endoscopy *n* = 191	Non-urgent endoscopy *n* = 265	*P*-value
Age (year)	53.0 ± 12.8	53.1 ± 11.4	0.88
Male, (%)	143 (74.9)	190 (71.7)	0.452
Hematemesis, *n* (%)	175 (91.6)	201 (75.8)	<0.001
Etiology of cirrhosis	–	–	0.43
HBV, *n* (%)	119 (62.3)	163 (61.5)	–
Alcohol, *n* (%)	12 (6.3)	26 (9.8)	–
HCV, *n* (%)	4 (2.1)	5 (1.9)	–
Schistosome, *n* (%)	8 (4.2)	9 (3.4)	–
HBV + alcohol, *n* (%)	14 (7.3)	9 (3.4)	–
Biliary, *n* (%)	7 (3.7)	14 (5.23)	–
Other, *n* (%)	27 (14.1)	39 (14.7)	–
Liver cancer, *n* (%)	36 (18.8)	41 (15.5)	0.342
Infection, *n* (%)	37 (19.4)	52 (19.6)	0.947
Portal vein emboli, *n* (%)	31 (16.2)	40 (15.1)	0.741
Ascites, *n* (%)	132 (69.1)	162 (61.1)	0.079
Hepatic encephalopathy, *n* (%)	25 (13.1)	30 (11.3)	0.567
Systolic blood pressure (mmHg)	113.1 ± 19.9	114.4 ± 17.7	0.18
Heart rate (beat/min)	88.1 ± 15.6	81.7 ± 15.2	<0.001
Laboratory values	–	–	–
TBIL (μmol/L)	29.2 ± 22.5	26.3 ± 20.5	0.114
ALB (g/L)	31.5 ± 5.8	33.3 ± 5.8	<0.001
PT (s)	15.6 ± 3.0	14.6 ± 2.0	<0.001
INR	1.4 ± 0.2	1.3 ± 0.2	<0.001
FIB (mg/dl)	1.3 ± 0.6	1.5 ± 0.7	0.114
PLT	82.7 ± 54.7	92.4 ± 60.2	0.078
Hb (g/L)	82.0 ± 22.5	84.0 ± 22.4	0.503
Therapy	–	–	<0.001
EVL	140 (73.3)	118 (44.5)	–
EIS + EITG	33 (17.3)	106 (40)	–
EVL + EIS + EITG	11 (5.8)	33 (12.5)	–
EIS or EITG	7 (3.7)	8 (3.0)	–
Antibiotic, *n* (%)	154 (80.6)	218 (82.3)	0.657
CTP score	7.8 ± 1.8	7.2 ± 1.6	0.001
MELD score	10.6 ± 4.0	9.3 ± 4.1	0.003
Glasgow-Blatchford score	12.4 ± 3.2	11.5 ± 3.5	0.004

HBV, hepatitis B virus; HCV, hepatitis C virus; TBIL, total bilirubin; ALB, albumin; PT, prothrombin time; INR, international normalized ratio; FIB, fibrinogen; PLT, platelets; Hb, hemoglobin; EVL, endoscopic variceal ligation; EIS, endoscopic injection sclerotherapy; EITG, endoscopic injection of tissue glue; CTP, child-turcotte-pugh; MELD, model for end-stage liver disease.

**TABLE 2:** Baseline characteristics of patients in urgent (≤12 h) and non-urgent (>12 h) endoscopy after propensity score matching (PSM).

Characteristics	Urgent endoscopy *n* = 133	Non-urgent endoscopy *n* = 133	*P*-value
Age (year)	53.7 ± 12.4	52.7 ± 11.9	0.51
Male, (%)	96 (72.2)	99 (74.4)	0.678
Hematemesis, *n* (%)	117 (88)	114 (85.7)	0.586
Etiology of cirrhosis	–	–	0.919
HBV, *n* (%)	93 (69.9)	84 (63.2)	–
Alcohol, *n* (%)	10 (7.5)	12 (9)	–
HCV, *n* (%)	2 (1.5)	2 (1.5)	–
Schistosome, *n* (%)	3 (2.3)	6 (4.5)	–
HBV + alcohol, *n* (%)	5 (3.8)	6 (4.5)	–
Biliary, *n* (%)	5 (3.8)	5 (3.8)	–
Other, *n* (%)	15 (11.3)	18 (13.5)	–
Liver cancer, *n* (%)	27 (20.3)	22 (16.5)	0.429
Infection, *n* (%)	23 (17.3)	28 (21.1)	0.436
Portal vein emboli, *n* (%)	21 (15.8)	22 (16.5)	0.868
Ascites, *n* (%)	87 (65.4)	90 (67.7)	0.697
Hepatic encephalopathy, *n* (%)	15 (11.3)	16 (12)	0.848
Systolic blood pressure (mmHg)	114.1 ± 19.6	113.4 ± 17.7	0.934
Heart rate (beat/min)	86.4 ± 13.8	86.0 ± 16.1	0.698
Laboratory values	–	–	–
TBIL (μmol/L)	27.4 ± 18.9	26.4 ± 17.3	0.757
ALB (g/L)	32.4 ± 5.3	32.4 ± 5.7	0.775
PT (s)	14.9 ± 1.8	15.0 ± 2.1	0.866
INR	1.3 ± 0.2	1.3 ± 0.2	0.89
FIB (mg/dl)	1.4 ± 0.7	1.4 ± 0.6	0.849
PLT	83.9 ± 51.5	83.3 ± 49.6	0.724
Hb (g/L)	84.2 ± 22.6	82.3 ± 22.0	0.388
Therapy	–	–	0.433
EVL	86 (64.7)	86 (64.7)	–
EIS + EITG	33 (24.8)	38 (28.6)	–
EVL + EIS + EITG	11 (8.3)	5 (3.8)	–
EIS or EITG	3 (2.3)	4(3.0)	–
Antibiotic, *n* (%)	108 (81.2)	108 (81.2)	1
CTP score	7.4 ± 1.6	7.4 ± 1.7	0.948
MELD score	10.1 ± 3.6	9.9 ± 3.8	0.74
Glasgow-Blatchford score	12.0 ± 3.2	12.1 ± 3.3	0.837

HBV, hepatitis B virus; HCV, hepatitis C virus; TBIL, total bilirubin; ALB, albumin; PT, prothrombin time; INR, international normalized ratio; FIB, fibrinogen; PLT, platelets; Hb, hemoglobin; EVL, endoscopic variceal ligation; EIS, endoscopic injection sclerotherapy; EITG, endoscopic injection of tissue glue; CTP, child-turcotte-pugh; MELD, model for end-stage liver disease.

### Clinical outcomes

**FIGURE 1:**
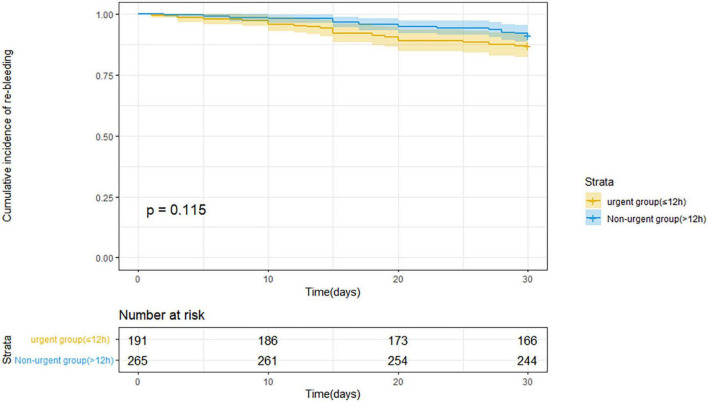
Kaplan–Meier estimates of 30 days rebleeding in patients with acute variceal bleeding before propensity score matching.

**FIGURE 2:**
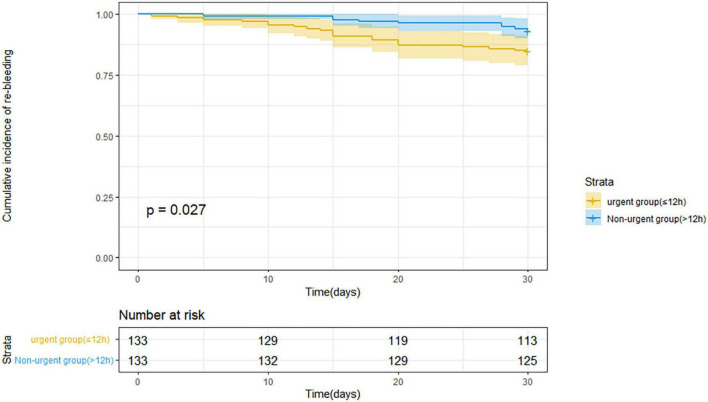
Kaplan–Meier estimates of 30 days rebleeding in patients with acute variceal bleeding after propensity score matching.

Table 3 indicates the clinical outcomes before PSM in both groups. The 30 days rebleeding rate was higher in patients who received urgent endoscopic treatment than in those who received non-urgent endoscopic treatment, but the difference was not statistically significant (13.1 vs. 8.7%, *p* = 0.13). The difference in the Kaplan–Meier estimates was also not statistically significant (*p* = 0.115) in Figure 1. The total number of transfused red blood cell units (2.4 ± 3.0 U vs. 1.6 ± 2.9 U, *p* < 0.001) and the number of transfusion products (1.5 ± 1.4 time vs. 1.2 ± 1.6 time, *p* = 0.001) in the urgent endoscopy group were significantly more than those in the non-urgent endoscopy group (*p* < 0.05). From Table 4, the 30 days rebleeding rate was significantly higher in the endoscopy group within 12 h after PSM than over 12 h (15 vs. 6.8%, *p* = 0.03). Similarly, the Kaplan–Meier method estimated a statistically significant difference in the 30 days rebleeding rate between the two groups in Figure 2 (*p* = 0.027).

**TABLE 3:** Clinical outcome of patients in urgent (≤12 h) and non-urgent (>12 h) endoscopy before propensity score matching (PSM).

Outcomes	Urgent endoscopy *n* = 191	Non-urgent endoscopy *n* = 265	*P*-value
**Primary outcome**			
Rebleeding, *n* (%)	25 (13.1)	23 (8.7)	0.130
**Secondary outcomes**			
Death, *n* (%)	9 (4.7)	9 (3.4)	0.477
Salvage treatment, *n* (%)	24 (12.6)	22 (8.3)	0.136
Number of units transfused (U)	2.4 ± 3.0	1.6 ± 2.9	<0.001
Number of blood transfusion products (time)	1.5 ± 1.4	1.2 ± 1.6	0.001
Length of stay in hospital (d)	9.3 ± 3.0	9.0 ± 3.3	0.087

**TABLE 4:** Clinical outcome of patients in urgent (≤12 h) and non-urgent (>12 h) endoscopy after propensity score matching (PSM).

Outcomes	Urgent endoscopy *n* = 133	Non-urgent endoscopy *n* = 133	*P*-value
**Primary outcome**			
Rebleeding, *n* (%)	20 (15)	9 (6.8)	0.03
**Secondary outcomes**			
Death, *n* (%)	4 (3)	5 (3.8)	0.736
Salvage treatment, *n* (%)	15 (11.3)	11 (8.3)	0.409
Number of units transfused	2.1 ± 2.4	1.9 ± 3.2	0.079
Number of blood transfusion products (time)	1.3 ± 1.3	1.4 ± 1.7	0.844
Length of stay in hospital (d)	9.2 ± 3.0	9.2 ± 3.0	0.819

### Predictive factors of the composite outcome of 30 days rebleeding and mortality

The results of the logistic regression analysis for the composite outcome are shown in Table 5. Univariate analysis suggested that the following parameters were significantly associated with 30 days rebleeding and mortality in EGVB patients with cirrhosis: age (OR: 1.04, 95% CI: 1.01–1.06, *p* = 0.002), liver cancer (OR: 0.51, 95% CI: 0.27–0.97, *p* = 0.039), shock on admission (OR: 0.27, 95% CI: 0.14–0.54, *p* < 0.001), CTP score (OR: 1.29, 95% CI: 1.11–1.50, *p* = 0.001), MELD score (OR: 1.11, 95% CI: 1.04–1.09, *p* = 0.002), and Glasgow-Blatchford score (OR: 1.11, 95% CI: 1.02–1.21, *p* = 0.016). Age (OR: 1.03, 95% CI: 1.01–1.06, *p* = 0.009) and shock at admission (OR: 0.31, 95% CI: 0.14–0.67, *p* = 0.003) were independent risk factors for the composite outcomes in EGVB patients with cirrhosis by multivariate analysis.

**TABLE 5:** Univariate and multivariate logistic regression analyses of the composite outcome of 30 days rebleeding and mortality.

Variable	Univariable OR (95% CI)	*P*-value	Multivariable OR (95% CI)	*P*-value
Timing of endoscopy	1.52 (0.88–2.61)	0.130	–	–
Age	1.04 (1.01–1.06)	0.002	1.03 (1.01–1.06)	0.009
Sex	1.15 (0.62–2.15)	0.652	–	–
Hematemesis	0.85 (0.43–1.68)	0.639	–	–
Etiology of cirrhosis	–	0.970	–	–
Liver cancer	0.51 (0.27–0.97)	0.039	0.59 (0.30-1.15)	0.122
Infection	0.58 (0.31–1.07)	0.080	–	–
Portal vein emboli	0.93 (0.45–1.93)	0.849	–	–
Ascites	0.67 (0.37–1.21)	0.182	–	–
Hepatic encephalopathy	0.76 (0.35–1.65)	0.489	–	–
Shock on admission	0.27 (0.14–0.54)	<0.001	0.31 (0.14-0.67)	0.003
Therapy	–	0.297	–	–
Antibiotic	0.63 (0.29–1.39)	0.254	–	–
CTP score	1.29 (1.11–1.50)	0.001	1.09 (0.89–1.33)	0.393
MELD score	1.11 (1.04–1.09)	0.002	1.07 (0.98–1.16)	0.134
Glasgow-Blatchford score	1.11 (1.02–1.21)	0.016	1.03 (0.94–1.13)	0.583

CTP, child-turcotte-pugh; MELD, model for end-stage liver disease.

## Discussion

The choice of when to perform endoscopy in patients with acute variceal bleeding in cirrhosis has been controversial. Some guidelines recommend endoscopy within 12 h of admission, while the Chinese guidelines recommend endoscopy within 12–24 h of admission. However, there is less evidence from actual clinical studies to support this (14). Therefore, we conducted a retrospective clinical study with a relatively large sample size to evaluate the relationship between the timing of endoscopy and clinical outcomes in EGVB patients with cirrhosis.

To date, several studies have reported on the optimal timing of endoscopic treatment for EGVB in cirrhosis. Two retrospective studies and one meta-analysis demonstrated that the timing of endoscopic treatment had no significant impact on clinical outcomes or prognosis of acute variceal bleeding (10, 11, 15). However, two clinical studies by Hsu et al. (16) and Chen et al. (12) with different time frames have reported that urgent endoscopic treatment was beneficial for the prognosis of patients with bleeding. On the contrary, Huh et al. (13) in a South Korean hospital in 2019 suggested that urgent endoscopy was not conducive to the prognosis of bleeding patients (34.4 vs. 19.1%, *p* = 0.005). Thus, studies of acute venous hemorrhage in cirrhosis remain small in number and heterogeneous, yielding highly biased results.

In this study, the baseline information of 456 patients included varied considerably due to the complexity of the actual clinical setting, which would have prevented further implementation of the study. Therefore, we used PSM to overcome this baseline imbalance by performing a 1:1 nearest-neighbor matching between the urgent and non-urgent endoscopy groups. Compared to previous studies, we attempted to adjust for this by taking into account more potential confounders in performing PSM. After PSM, there was no significant difference in baseline information between the 133 paired patients with EGVB.

We need to consider the selection of appropriate outcome indicators when it comes to studies related to endoscopic therapy. Previous studies have focused on outcomes such as rebleeding at 6 weeks, mortality, or a combination of both. Moreover, according to the Baveno VII workshop, 6 weeks mortality should be the primary endpoint for studies on the treatment of acute variceal bleeding (8). Early evidence suggests that patients who do not receive prophylactic treatment after their first variceal bleeding have a rebleeding rate of 60% and a mortality rate of 33% within 1–2 years (17). Therefore, the determination of the specific prognosis should be closely related to the timing of secondary prevention of variceal bleeding. The Chinese guidelines recommend that patients whose varicose veins have not completely healed after the first endoscopic treatment are generally scheduled for follow-up within 1–3 months (7). In our hospital, the second endoscopic treatment was performed after 30 days of follow-up for patients with variceal bleeding, thus, the outcome measures were defined as 30 days rebleeding and mortality.

Our results are similar to the Korean study (13). Although the shorter 30 days follow-up resulted in a significantly lower incidence of outcomes, there was a significant difference in the 30 days rebleeding rate between the two groups (13.1 vs. 8.7%, *p* = 0.03). Some possible explanations for the high rate of 30 days rebleeding with urgent endoscopy are as follows. First of all, urgent endoscopy prevents the residual food and blood in the stomach from emptying promptly, which can obscure the endoscopist’s view and result in much lower surgical outcomes. Second, a lack of basic resuscitation therapy or short resuscitation time in patients with peripheral circulatory collapse after bleeding may lead to reduced tolerance during endoscopic procedures, affecting the quality of the procedure and postoperative recovery (18, 19). In addition, urgent endoscopy may give rise to inadequate use of medications to reduce portal pressure and acid suppression. Furthermore, excessive portal pressure increases the likelihood of hemostatic failure. Although there is no evidence that early use of PPI affects the clinical outcome of patients, the success rate of hemostasis can be improved when gastric fluid pH is >5 (7, 20). While we should be cautious in interpreting these results, the 12 h completion of urgent endoscopy recommended by some current guidelines may not be the best option for all patients with EGVB, especially those in whom fluid resuscitation is urgently needed to restore blood volume. However, our results also found no significant difference in 30 days mortality between the two groups, which is similar to the results of Yoo et al. (11) but non-urgent endoscopy had a higher mortality rate. This may be limited to a statistically insignificant result due to insufficient sample size, which subsequently needs to be verified in a prospective multicenter study with a large sample. In brief, the choice of the appropriate time for endoscopy depends on the current progression of each patient’s condition and the local level of care.

In this study, regression analysis of candidate risk factors for EGVB identified advanced age, hepatocellular carcinoma, shock on admission, CTP score, MELD score, and Glasgow-Blatchford score as risk factors for the composite outcome of rebleeding and mortality at 30 days. To the best of our knowledge, most studies have shown that poor prognosis is related to advanced age, the severity of cirrhosis (CTP grade, MELD grade, etc.), comorbidities (hepatocellular carcinoma, portal vein thrombosis, etc.), and treatment modality (21–24). The risk factors considered by different studies varied slightly, which may be related to different study populations and inclusion criteria. The results of our analyses revealed that age and shock at admission were independent predictors of the composite outcome. The natural aging of elderly patients causes multiple organ function decreases, multiple comorbidities can impair organ function, and tolerance to bleeding shock is significantly lower than that of younger patients. Even if the shock is aggressively corrected, hypoperfusion can directly or indirectly damage vital organs in the body, which may lead to the occurrence of sequelae. For acute bleeding, the hemostasis rate of EVL can reach from 90 to 95% and can effectively reduce the rate of rebleeding (25). This study found that patients with shock were more likely to use combination therapy (EVL + EIS + endoscopic injection of tissue glue) than those without shock (14.89 vs. 9.05%). Findings may vary in clinical studies in different populations and treatment settings, but we should actively intervene to improve poor prognoses and outcomes.

This study has some limitations. First, this is a single-center, retrospective clinical study with possible selection bias. Second, clinical outcomes are observed and followed up for a short period, which does not fully reflect the long-term prognosis and the occurrence of adverse events. In addition, the sample size of this study was small and needs to be further expanded to improve the accuracy and credibility of the study. Despite these limitations, this is the first study to correct for more confounding factors, observing a shorter period, for poorer outcomes in EGVB.

In conclusion, the time for endoscopy is a significant indicator of 30 days prognosis in patients with cirrhotic EGVB. The urgent endoscopy group (≤12 h) is significantly associated with a poorer rebleeding rate in patients with EGVB, whereas time for endoscopy is not associated with mortality. In future, we plan to conduct a prospective study to validate this more accurate timing of endoscopy.

## Data availability statement

The raw data supporting the conclusions of this article will be made available by the authors, without undue reservation.

## Ethics statement

The studies involving human participants were reviewed and approved by the Human Ethics Committee of The First Affiliated Hospital of Nanchang University [(2022)CDYFYYLK(11-034)]. The patients/participants provided their written informed consent to participate in this study. Written informed consent was obtained from the individual(s) for the publication of any potentially identifiable images or data included in this article.

## Author contributions

KW organized the database and wrote the first draft of the manuscript. YF, ZG, and XZ wrote sections of the manuscript. All authors contributed to the conception, design of the study, performed the statistical analysis, manuscript revision, read, and approved the submitted version.
